# How Do Medical Students and Doctors in the UK Health System Reach Decisions About Specialty Choices Through Their Undergraduate and Postgraduate Training?

**DOI:** 10.7759/cureus.98263

**Published:** 2025-12-01

**Authors:** Amy Fielding, Christopher Williams

**Affiliations:** 1 Leicester Medical School, University of Leicester, Leicester, GBR

**Keywords:** choice of medical specialisation training, effect of covid-19 on medical students, general practice trainee, hospital-based medicine, junior doctor, medical career, medical specialty, nhs workforce, work-life balance, medical students

## Abstract

Background

The process of making a specialty choice is one of the most important decisions of a doctor’s career. This choice is dynamic and multifaceted, with important contributing decisions made at various career stages. To understand the complexities behind these career decisions and help target recruitment of undersubscribed specialties, we must understand the influences on specialty choice and how these influences change as careers progress, from undergraduate to fully qualified doctors. This research aimed to define what influences these decisions, and uniquely denote how these influences evolve as trainees’ careers progress, by investigating three components: what decision-making processes do participants use at different times, how they weigh different factors and influences, and does the importance of these factors and influences changes at different points.

Methodology

The data for this naturalistic qualitative research project were collected using semi-structured interviews, with nine participants spanning a continuum of career stages, from final-year medical students to doctors who had completed a Certificate of Completion of Training (CCT; hereafter referred to as post-CCT doctors). We analysed the data thematically and used the data gathered to produce a conceptual model.

Results

This research demonstrated that, as time progresses within careers and trainees gather more experience, their reliance on stereotypes and other wider social narratives reduces, while the influence of wider social roles increases. This model also hypothesises four phenotypes of specialty decision-making: early committers, re-thinkers, repeated re-committers, and late committers. Other themes of influencing factors included interest in the specialty, placement experience, and gender.

Conclusions

These findings show the complexity of factors affecting specialty choice and illustrate the dynamic nature of the decision. The variance of specific influences with age, such as wider social narratives, indicates implications for both medical schools and the Royal Colleges with regard to both support and recruitment.

## Introduction

Current UK training structures

The phenomenon of specialty choice has been studied but remains incompletely understood. Medical training begins as an undergraduate (with or without a previous degree), requiring ‘four to six years of study’ [1]. As graduates, they then enter a two-year ‘Foundation Programme’ rotating across six specialties as resident doctors. Midway through the second year, they are expected to make initial specialty training applications. These are subject to a national ranking process to guide the allocation of jobs. There is an option to pause training at this point and take a Foundation Year 3 (FY3), which is typically used to gain ‘more experience in specialties’ or ‘build (your) portfolio’ [2].

The next stage depends on the chosen specialty. Specialist Consultant training within National Health Service (NHS) hospitals can be ‘run through’ (appointed to Specialty Training year 1 posts (ST1) and continuing through to ST8) or ‘uncoupled’ (where the programme is divided into two stages: core training and higher specialty training), whereas General Practice (GP) training consists of ‘three years of approved clinical training’ [3]. Upon completion of their training pathway, trainees receive a Certificate of Completion of Training (CCT) (subject to satisfactory completion of all training elements, including postgraduate qualifications).

Historical comparisons

In 2005, the NHS medical training pathway underwent reform, under the title of ‘Modernising Medical Careers’ (MMC) [4]. These reforms were driven by a report produced by the Department of Health in the year 2000 [5], which indicated a policy aim to streamline postgraduate medical training to achieve a ‘30% expansion in consultant numbers with further increases in the pipeline’ by 2004. The reform incorporated five key principles, including ‘time-limited’ and ‘programme-based’ training [6].

The impact of this change left many junior doctors unemployed, and after a subsequent inquiry led by Sir John Tooke in 2008 [7], adjustments were made. As opposed to the previous structure of three to four years of rotations before deciding, doctors now have 1.5 years from graduation to apply for specialties. Svirko et al. [8] studied graduates’ opinions of the specialty application process after the reform and found that ‘the majority of the post-MMC cohort said that they had to make their specialty decision too early’. Reflecting this view, Church et al. [9] noted the trend towards ‘FY3 years’ being undertaken: ‘since 2017, more than 50% of UK doctors have undertaken a “Foundation 3 (F3) Year”, which they partly attributed to a ‘lack of preparedness’ for entering specialised training programmes.

Models for making career choices

Several attempts have been made to develop models of how individuals make these decisions. A common feature of many of the models produced is that there are multiple stages, beginning with ‘Self Evaluation’. In the ‘DOTS model’ [10], the participants are encouraged to undergo ‘opportunity exploration’ before ‘making a decision and ‘transitioning’. This model demonstrates the process doctors go through between foundation years and specialty training; however, specificity on how these choices are made is limited. These influences are described more thoroughly within the Bland-Meurer model [11], which defines how medical students make their specialty choice and suggests that ‘students will try to match the perceived characteristics of the specialty to their career’ [12]. This demonstrates that career choice is a process and that students are influenced by their medical school (characteristics and curriculum type, for example), their assessment of their own personal characteristics and lifestyle, and their perceptions of the specialty choices, to formulate their decision.

Influences on career choice

Singh et al. [13] explored why UK medical students change careers in an interview study. The study highlighted important themes, including the influence of clinical teachers, the curriculum, and clinical. However, only medical students who were sure about their specialty choice before changing their mind were included, and the extent to which it can be extrapolated to other careers is uncertain.

Mahoney et al. [14] conducted a prospective longitudinal study building on the previous work by McParland et al. [15] comparing the career intentions of a cohort of second-year medical students with their intentions as a senior house officer. They found that ‘a minority of students who had stated a definite career choice gave the same “definite” response for that specialty after qualification’. This is reflected in earlier research by Lambert et al. [16], which showed that seven years after graduation, only 36% of doctors had entered their initial career choice. Importantly, both studies were conducted before the medical training reform.

Specific factors associated with specialty choice

Gender

Gender in medicine is increasingly discussed, and, in recent years, there has also been interest in the role that gender plays in influencing specialty choice. Woolf et al. [17] found that women were more likely to apply to general practice, obstetrics and gynaecology, and paediatrics, and men were more likely to apply to core surgical training, acute care/emergency medicine, and clinical radiology. A common emphasis is on the barriers faced by women within certain specialties. For example, a study [18] concluded that ‘women are less likely to enter and complete surgical training’.

Specialty Prestige

‘Generalism is associated with lower prestige in the medical profession is already present at the very start of medical school and seems to be reinforced during undergraduate training’ [19]. Norredam et al. [20] linked this to recruitment: ‘Low-status specialties have more difficulties recruiting qualified personnel’. It must be appreciated here that this literature is potentially outdated, particularly considering the change in specialty roles and redeployment during and after the pandemic. Furthermore, they are not UK-based studies.

Aims and objectives

From existing literature, specialty choice unfolds over an extended period of time and is influenced by a wide range of factors (including those mentioned in previous studies). Amalgamating this information, we formulated three key aims for this research: to establish the decision-making processes used in specialty choice, to understand how participants weigh different factors and influences, and to explore how the importance of these factors evolves over time.

## Materials and methods

A qualitative design method involving thematic analysis was chosen for this project. This allows a naturalistic lens to reach a deeper understanding of individuals’ lived experiences and allows significant participant freedom to respond to prompts and questions. A constructivist perspective was adopted, with an inductive approach to analysis, allowing themes to emerge from interpretation of the data.

Postgraduate participants were located in the NHS, in the Midlands region of the UK. Undergraduates were sampled from Leicester Medical School. Nine participants were interviewed. We chose to interview nine participants, as this number was the maximum feasible within the timeframe of our project, and, after discussion with my supervisor, it was felt likely to allow adequate exploration of the research questions. We also decided that we wanted to incorporate a variety of career stages to understand and explore themes and how they change as careers progress. From the literature, we identified three appropriate transition points between career stages to shape our purposive sample and sampled three subgroups of equal numbers. The three key transition points between career stages were identified as: final-year medical students, middle-grade doctors (in training), and post-CCT doctors (including one Medical Consultant, one Surgical Consultant, and one General Practitioner). The details of the participants can be found in Table 1.

**Table 1:** Participant characteristics. CCT = certificate of completion of training (consultant); FY3 = foundation year 3; ST2 = specialty training year 2; ST4 = specialty training year 4

Participant number	Gender	Ethnicity	Stage of training	Specialty (chosen or desired)
1	Male	White British	Final-year medical student	Surgery
2	Female	White British	Final-year medical student	Undecided
3	Female	British Asian	Final-year medical student	Paediatric Surgery
4	Female	White British	Post-CCT	General Practice
5	Female	British Asian	FY3	General Practice
6	Female	White British	ST2	Emergency Medicine
7	Male	British Asian	ST4	Emergency Medicine
8	Male	White British	Post-CCT	Microbiology
9	Male	White British	Post-CCT	Urology

The interviews took place virtually over Microsoft Teams (Microsoft® Corp., Redmond, WA) [21], led by the lead author, and were of a semi-structured nature, with the use of a pre-prepared interview guide, which was used flexibly to explore themes as they surfaced. Each participant answered adapted questions based on their career stage. The interview guide was formulated using the study aims, using insights from our literature review, and the gaps noticed within the field to build on them. We chose to use a semi-structured method of interviewing as the topic of interest was complex and multifaceted, which ensured that the aims of the study were achieved, while still allowing conversational discourse. All interviews were recorded on an encrypted device and uploaded to a university-managed computer for manual transcription.

Reflexive thematic analysis, theorised by Braun et al. [22], was used to explore the results. Thematic saturation was ensured by completing iterative analysis. Coding was completed manually, without the use of software. Individual codes were grouped into overarching categories and then themes. We engaged in reflexive discussions about the codes, critically examining how our backgrounds and experiences may have influenced our interpretations of the data. Furthermore, records of analytic decisions were kept to ensure transparency. A narrative description of each theme was then prepared, noting relationships across them. As a final analytical step, the outputs of the thematic analysis were triangulated with other existing literature to create a conceptual model of career-related decision-making.

## Results

The thematic analysis produced five overarching themes: interest in the specialty, placement experience, stereotypes, gender, and work-life balance.

Interest in a specialty

Across all training stages, interest in the specialty was an important driver of specialty choice. It was felt important to be in a specialty that was interesting, both in terms of its subject matter and the procedures, tasks, and routines of that specialty. As other influences were discussed, there was a sense in which these were balanced against interest in specialty in reaching a decision: ‘You don’t want to be in something you dislike, just because it affords a better lifestyle’ (Participant 7).

Placement experience

We found that participants’ experience during clinical placements emerged as an important influence, discussed under five sub-themes: supervisors, colleagues, and working environment; timing of placement; placement location; and the COVID-19 pandemic.

The influence of an enthusiastic supervisor, mentor, or other senior role model within the specialty was important, with a majority stating that they were more likely to be interested in pursuing a specialty if they had this experience. Another participant approached this from another perspective, from his own experience of taking on the role of the supervisor, and, as a result, increasing the number of students interested in pursuing his specialty.

Some participants attached further importance to colleagues actively showing the specialty as a potential career path, acting as ‘ambassadors’ for the specialty. This is more of an act of promotion rather than a more human act of support when they were inexperienced within the specialty. Equally, when these figures were lacking, some participants were disinclined to consider the specialty any further: ‘No one was really that bothered’; therefore, ‘I had no interest in doing them’ (Participant 7).

Besides personal experience, this study found that interviewees (particularly postgraduates) were influenced by the experience of friends and family in various specialties, and this was one way in which career choices appeared to be socially mediated. One participant discussed how a family member’s struggle in the pursuit of a specialty that she had been strongly considering ultimately deterred her. This was echoed by others who discussed how they looked on as colleagues pursued specialties and later changed their direction. This acted as an influencing factor in their own choices.

Placement location

Location of the placement experience, especially for undergraduate placements, was an important factor. Participants highlighted the distinction between experiencing a specialty within a large university teaching hospital or at a district general hospital (DGH). In DGHs, students tended to feel more immersed within the specialty environment due to smaller numbers of students on placement, and this influenced their willingness to consider a specialty. However, the disadvantage of DGH placements for some participants was a perceived lack of exposure to key elements of the specialty. This emerged in emergency medicine, where participants felt that they were disadvantaged by being at a smaller unit.

COVID-19 pandemic

The COVID-19 pandemic was an important influence on participants in various specialties, but its influence varied at different stages of training. Final-year medical students discussed their limited impact on their perceptions of specialties, as they had experienced many of these specialties before the pandemic in earlier clinical years. Several of the junior doctor participants had remained in a particular job for double the length of time of a standard specialty rotation. For some, such as Participant 6, this led them towards a specialty, as they felt like ‘part of the furniture’; in others, they were discouraged from pursuing it.

Stereotypes

We discovered that the concept of 'stereotypes’ was another key factor in decision-making. This term was used by participants to refer to opinions that were formed not from direct experience (or experience of those within their own social network) but which were well-recognised, shared, and discussed within the medical profession.

Awareness of these stereotypes appeared highly prevalent: all participants recognised that they knew of specialty stereotypes, but that they were not always accurate. Some specialties appeared to have deeply ingrained stereotypes. For example, participants (without specific prompting) described a stereotypical orthopaedic surgeon in similar terms to Participant 9: ‘Alpha male who’s very interested in fixing the bones and nothing else’. Importantly, ‘stereotypes’ referred not just to practitioners but also to the specialty itself and what was required to succeed, particularly in reference to the academic, ‘work-oriented nature of ‘surgical specialties’. Decision-making was more affected by an interaction of stereotypes and the person’s ‘sense of self’, defined as the 'feeling of identity, uniqueness, and self-direction’ [23]. This shaped the person’s perception of whether they could be successful in the specialty.

Gender

The influence of gender on specialty choice was discussed extensively by participants. Among participants who identified as male, there was a sense of having been at an advantage. A common experience among female participants was to have experienced or witnessed negative comments related to their gender, particularly in references to conversations with senior male surgeons on placement, such as ‘my advice to you is, if you want to have a family, don’t do surgery’ (Participant 3). Participants felt that these experiences would not have stopped them from pursuing the specialty but would have been a contributory factor to their decision-making.

A particular concern among female interviewees was the impact of maternity leave on their training and the disadvantage of time out on clinical skills, such as in surgery. Similar reflections were shared by another female participant who talked about her own career progression being slowed due to maternity leave, and socially mediated experiences of slower career progression having contributed to decisions to change specialties with shorter training durations.

Less than full-time training

Among postgraduates, the majority of interviewees were either currently working part-time or were considering moving to this pathway within the next year. Perceived openness within the specialty to less-than-full-time working was a key theme, with one participant speaking of their attraction to emergency medicine due to its perceived openness to flexible working.

General practice

The appeal of GP was discussed, unprompted, with every participant. All female interviewees working at the postgraduate trainee level were either currently training in primary care or were strongly considering it as a future option. There was an interaction between the interest in the specialty and other broader lifestyle benefits of choosing GP as a career. Participant 6, currently on a different training pathway, added that primary care remained a ‘fallback’ option.

Personal life

The compatibility of specialty choice with family life was a factor discussed by all but one participant, who was an undergraduate student. Besides perceived openness to flexible working, other relevant factors included the impact of out-of-hours work.

Three participants, all of whom identified as female, reflected on the relevance of their being in a relationship with a ‘hospital medic’, and the compatibility of their career choice with that of their partner was discussed. For example, one undergraduate participant who was hoping to pursue surgery was in a relationship with another aspiring surgeon and discussed the difficulties of specialty decisions. Within this sample, female participants had switched to community medicine to avoid issues with family life.

Increasing importance of work-life balance

The importance of work-life balance appeared to increase for participants as they moved from undergraduate to postgraduate training. Most undergraduates felt that work-life balance was not a priority for them. Correspondingly, every postgraduate doctor acknowledged work-life balance as a priority and recognised that this had changed from their earlier careers: ‘Coming to the end of F2, the importance of being able to balance life was becoming greater’ (Participant 7).

In summary, the themes found span educational, practical, and personal contributing factors to specialty choice. It is clear that the experience within a specialty encompassed a wide range of specific influences, from all stages of participants.

## Discussion

Influences on specialty choice

Our research demonstrated several themes that align with current literature, particularly the most recent study produced by Singh et al. [13], but this study provides several new insights. Trainees obtained the information used to shape their specialty choice from three sources: personal experience in the specialty; the opinions of family, friends, and colleagues; and wider social narratives.

An important theme within this data was the impact of experience in the specialty, at all levels. At the undergraduate level, students were most influenced by how much staff involved them, likely because they have an observer role and require a doctor to facilitate (to a varying extent) their involvement. Resident doctors were more influenced by the support they received within a specialty from all staff. This study adds findings on the aspects of the clinical experience that play a role, including location and timing. Timing of clinical exposure is a novel finding, suggesting that the timing of a particular specialty placement relative to the student’s/doctor’s personal life impacts the likelihood of pursuing that specialty. With regards to the location of placements, this study suggests that DGHs have distinct benefits, but that consideration of the specialty and its availability at various locations should be given. Placements in a DGH within many of the medical and surgical specialties appeared to reap benefits in terms of trainee involvement; however, it compromised access to more specialist areas.

We discovered that experience in various areas secondary to the COVID-19 pandemic may have impacted specialty choice. If both students and doctors rely on experience within specialties to form career decisions, altered foundation rotations will have an impact, supporting Kotta et al.’s [24] prediction that ‘the number of doctors taking an FY3 rising as we come out of this pandemic’. It also adds to the criticisms of the MMC reformation’s move towards earlier commitment to specialty.

Another significant finding is the value that trainees put on the experiences of their colleagues. Doctors are likely to be swayed from their initial specialty choice if a close friend has a negative experience. This was reported most often regarding women’s experience in a placement and seemed to then impact other female colleagues. In this study, the most common examples mentioned by participants related to work-life balance and their other social roles, particularly those with families, which then dispels other female trainees who have intentions of a family. These were often expressed by participants in terms of ‘stereotypes’, with their influence spanning across all grades of trainees. These appeared to have a negative influence on trainees at the undergraduate level, whereas more senior postgraduates sometimes saw ‘stereotypes’ positively as a ‘group identity’ and a challenge to overcome.

Where direct specialty experience was lacking, particularly at the undergraduate level, participants based their decisions on the impression of close colleagues and wider social narratives (including ‘stereotypes’). This appraisal included an assessment of the ‘specialty environment’ and creating a ‘group identity’ of doctors working within it. During the pandemic, this appraisal process may have been hindered and resulted in students and resident doctors relying more on preconceived perceptions of specialties than direct experience.

Our results demonstrated that participants reflect on their sense of professional identity and self-direction, comparing this with their perception of what is required for the specialty. This was discussed by more than one participant, suggesting the power of stereotypes to induce the feeling of doubt and inadequacy (particularly among undergraduate students). This was also important as specialties with negative or prestigious stereotypes may be disadvantaged in terms of medical student recruitment.

Previous literature, such as the works by Creed et al. [25], has emphasised the importance of ‘specialty prestige’ to career decision-making. In this study, the desire to be in a ‘prestigious’ specialty did not appear to be a significant influencing factor.

This study identified three motivating factors, which included ‘Intrinsic Interest in the Specialty’, ‘Wider Social Roles’ and ‘Sense of Desired Professional Self’. Individuals’ views of these areas changed as they progressed through their undergraduate and postgraduate careers; for example, finding different interests or changing social roles. As individuals moved towards a specialty decision, they appeared to balance these motivating factors and compare them with their appraisal of the specialty. The relative importance of different motivators varied for different individuals, but their importance as factors in decision-making changed with time, with a greater weight for work-life balance among postgraduates.

We discovered that an interest in the medicine the specialty encompasses was the leading influence of specialty choice; however, this appears more dynamic than suggested in previous literature, such as the study by Singh and Alberti [13]. The general term of ‘innate interest’ as a career influence does not acknowledge the interplay between experience and interest, with some participants having ‘innate interest’ in a specialty and then going on to further explore that placement, where the experience either confirms or disconfirms their choice and vice versa. Therefore, while it is important to maintain ‘experience’ and ‘interest’ as discrete influences, the relationship between them should be recognised.

Work-life balance was a heavy influence on specialty. The interest in switching to ‘less than full-time training’ was evenly distributed between males and females, potentially reflecting a modernisation of gender roles. As this study captured individuals at multiple career stages, the evolution of the increasing importance of work-life balance as one progresses through training is demonstrated. This supports statistics produced by Goldacre et al. [26] about how desired specialty choice changes throughout their career, which is shown within this study as a participant strongly considered changing specialty, secondary to a work/life imbalance. In contrast to this study, more recent cohorts in the authors’ study showed gender equality in specialty change, whereas in this study, the majority of discussions regarding changing their specialty took place with female participants. Our sample size was not large enough to dispute this literature completely.

Gender as an influence on specialty choice was found repeatedly. Many females experienced resistance from male surgeons about the pursuit of a surgical career and received negative gender-based comments during training placements, influencing their own placement experience and the experience of their female colleagues and friends. While participants found these comments unwelcome, they felt able to ignore them and were not themselves deterred from pursuing this career pathway. The key influence of gender concerned wider social roles, regarding practical concerns about becoming a parent and its subsequent impact on training. Parental leave was recognised to require a break in training to varying extents, but female participants were more likely to feel a sense of responsibility to switch to part-time training when they have children, and therefore sought not only to increase their training length by working less-than-full time but also by choosing specialties which accommodated a better ‘work-life balance.’ In heterosexual relationships with another doctor, the female partner also appeared more likely to change course, particularly if working in the hospital setting. These findings challenge the work of Woolf et al. [17], who proposed the concept of gender-popular specialties. Even though some female participants are pursuing a career in GP, there were also female representatives for both acute care common stem and surgical specialties.

Decision-making phenotypes

From the research we have conducted, we have identified four broad decision-making phenotypes within our participant group regarding specialties: early committers, re-thinkers, repeated re-committers, and late committers. ‘Early committers’ include trainees who decide they want to train within a specialty early within undergraduate medicine and have pursued it. ‘Re-thinkers’ were most common within our data set, and this is supported in the literature, particularly within Mahoney et al’s [14] statistic of only 36% of trainees pursuing their original specialty choice. This describes those who decide on a specialty at the undergraduate level but then change infrequently and often after they begin practising. ‘Repeated re-committers’ were those who, at undergraduate and postgraduate levels, frequently changed their desired specialty, with the change driven by unexpected interest in a new specialty. ‘Late committers’ delay making a decision and describe themselves as ‘undecided’, making their decision close to applying for specialty training. This was most prominently seen in the senior participants within this study, which may coincide with previous postgraduate training structures. We acknowledge that there may be alternative categories defined in a larger participant group.

Conceptual model of specialty choice

**Figure 1:**
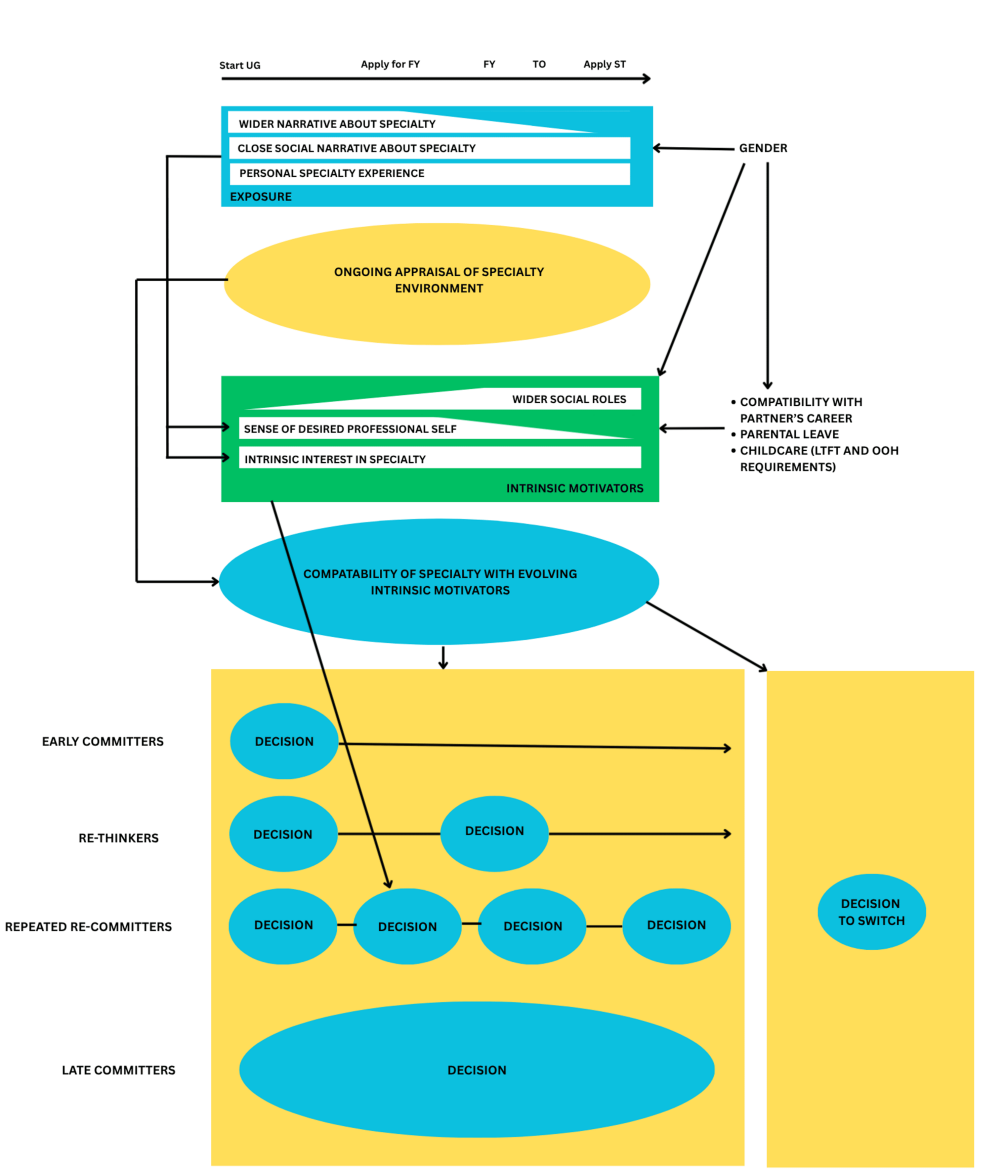
Conceptual model of specialty choice. The model depicts how social influences, personal experiences, and intrinsic motivators interact over time. The size of each wedge represents the changing importance of these factors along the longitudinal axis. Their evolving compatibility leads to four decision-making phenotypes: early committers, re-thinkers, repeated re-committers, and late committers. FY = foundation years; LTFT = less-than-full time; OOH = out of hours; ST = specialty training; TO = time out; UG = undergraduate

The conceptual model presented in Figure 1 was developed through an in-depth analysis of the themes that emerged from this project. These themes were then compared with and interpreted in the context of existing literature, resulting in a framework that is conceptually extrapolated beyond the immediate findings of the study. A key factor of this model, which differentiates it from the Bland-Meurer Model [11], is the time axis. Changes with time were found across all themes identified in this study; thus, the model includes this, and is labelled with key career decision points. It also includes interim points, such as ‘Time Out’ (TO), which occurs (in the UK context) in the context of an F3. The changing importance of different factors with time is reflected in the wedge shape for that influence within the model, with width reflecting its relative influence on specialty choice at that point. Thus, as careers progress, work-life balance becomes a larger influencing factor, while ‘Wider Narrative About Specialty’ and ‘Sense of Professional Self’ tend to have a reducing influence. This study suggests that the person’s level of interest in the specialty remains a consistent feature of decision-making as time progresses. Appraisal of the specialty environment appears to be an ongoing process among undergraduate and postgraduate trainees, with their perceptions changing as they experience new specialties. This links to the aim of ‘career exploration’ mentioned in the ‘DOTS Cycle’ [10]. This appraisal then contributes to an evolving understanding of compatibility for a specialty. This was a strong theme in this data and is similar to the ‘self-perception’ incorporated within the Bland-Meurer Model [11] as a process contributing to specialty choice. My model portrays appraisal as continual, reflecting experiences and intrinsic motivators as dynamic, thus compatibility must remain dynamic as well. The profound influence of gender has been added to the model and shown as a contributor to experience and intrinsic motivators. Gender has been shown to influence exposures, particularly in my data set, students’ experience on certain placements (female students being perceived negatively within surgery), and the wider and closer narratives, in terms of what society, and often family members, experience and expect from each gender role.

Strengths and limitations

The use of longitudinal in perspective sampling is a strength of this study, replicating the benefits of a longitudinal study. Another strength was using a literature search to help formulate the question guide, which gave structure to the interviews and allowed the themes cultivated during the study to be compared with existing literature. To acknowledge weaknesses in this study, although the use of various grades of doctors was important, some of the participants were graduate or mature entry students when they began their medical studies. This is a purposive sample from within the current NHS workforce; however, it does lead to conflicting influencing factors, particularly as graduate entrants may have different social roles early in their careers. It is important to acknowledge our potential biases as researchers within the profession. To ensure reflexivity, we engaged in regular discussions with our supervisor regarding interpretations and analysis of the data. Furthermore, the sample was based on trainees who had trained solely in the UK. Given that ’one in seven’ [27] employees of the NHS identify as a non-British nationality, this sample may not be representative of the multicultural workforce and could ignore cultural influencing factors on specialty choice. The relatively small participant size (n = 9) may limit the transferability of the findings; however, the aim of the study was to assess the depth of understanding, and importantly, this number was sufficient to achieve thematic saturation. Further research to support the study findings should be undertaken with a larger group of participants.

Implications

Our data highlighted implications for both medical schools and royal colleges. Particularly in surgical specialties, guidance on career paths was deemed poor; thus, it would be beneficial for medical schools to make clear career information available to allow for early preparation in competitive fields. A further theme in our data was the concept of role modelling, which could be utilised by medical schools appointing a representative doctor in that specialty, to be a point of contact for interested students. Furthermore, we would recommend an increased awareness of the location of placements to maximise specialty clinical experience, particularly for students who are considering a career in that specialty. The results of this study highlight implications for the Royal Colleges of various specialties, and, in particular, those who are experiencing a loss in recruitment/retention in the recent past. If positive experiences within a specialty can be provided, then this study suggests that this would have a positive impact on recruitment. This could be facilitated by opportunities for ‘experience placements’ within their specialty, through the Royal Colleges directly, which nulls the location limitations discussed in our themes. To further comment on recruitment, it appears from this study that many participants did not decide on a specialty until they were in postgraduate training, but their experiences in undergraduate contributed to their decision. It is appropriate to infer that the Royal Colleges could aim to spark interest in specialties at an undergraduate level.

## Conclusions

This study provides new insights into how UK medical trainees make decisions about specialty choice, highlighting the dynamic interplay between clinical experience, social influences, and personal motivations. Specialty exposure at both undergraduate and postgraduate levels was a key determinant, with the timing and location of placements shaping perceptions and engagement. Social narratives, including colleagues’ experiences and perceived stereotypes, also significantly influenced career decisions, particularly where direct specialty exposure was limited (such as during the COVID-19 pandemic). Motivating factors such as intrinsic interest in the specialty, work-life balance, and professional identity evolved over time, with gender and wider social roles playing a notable role in shaping specialty preference. The data shows that as time progresses within careers and trainees gather more experience, their reliance on stereotypes reduces, while conversely, the influence of wider social roles increases. The study has led to a conceptually extrapolated model, developed from the collected data, which illustrates how individuals appraise various influences and identifies different phenotypes of specialty decision-making: early committers, re-thinkers, repeated re-committers, and late committers. This research is particularly useful, not only from a recruitment perspective but also, and more importantly, to ensure that doctors can reach a specialty that lines up with their sense of self and wider commitments and keeps them engaged and fulfilled. This area of research is continuously evolving, as the dynamics of gender and social roles, and the training programmes themselves, change. Although this research has highlighted some key features of the decision-making process and elaborated on the theory behind it, more research is needed. It would be beneficial to research the interventions that could increase the recruitment/retention of undergraduates and doctors into specialty training. Another avenue of research would be a study on the reflections of senior doctors regarding career choices and influences, allowing the expansion of the findings of this study to encompass a whole career in medicine.
